# PREDICT v3.0 outperforms v2.2 in young (25–40 years) breast cancer patients according to real-world data from a large Swedish population-based registry

**DOI:** 10.1186/s13058-026-02271-2

**Published:** 2026-03-31

**Authors:** Christine Lundgren, Irma Frediksson, Antonios Valachis, Daniel Robert Smith

**Affiliations:** 1 Department of Biomedical and Clinical Sciences, Linköping University, Linköping, Sweden; 2 Department of Oncology, Region Jönköping County, Jönköping, Sweden; 3 Department of Breast, Endocrine Tumors and Sarcoma, Karolinska University Hospital, Stockholm, Sweden; 4 Department of Molecular Medicine and Surgery, Karolinska Institutet, Stockholm, Sweden; 5 Department of Oncology, Faculty of Medicine and Health, Örebro University Hospital, Örebro, Sweden; 6 Clinical Epidemiology and Biostatistics, Faculty of Medicine and Health, School of Medical Sciences, Örebro University, Örebro, Sweden; 7 The Institute of Cancer Research, London, UK

**Keywords:** PREDICT model, Young patients, Breast cancer, Prognosis

## Abstract

**Background:**

PREDICT is a widely used prognostic tool supporting treatment decisions in breast cancer care. As v3.0 was recently released, we performed an external validation of PREDICT v2.2 and v3.0 in young (≤ 40 years) breast cancer patients.

**Methods:**

We included Swedish non-metastatic breast cancer patients aged ≤ 40 years, diagnosed between 2008–2019, from a registry-based research database. Outcomes were all-cause and breast cancer-specific mortality (BCSM) at 5- and 10-years, in all and in prespecified patient subgroups (by molecular subtype and nodal status). Model calibration and discrimination were evaluated by the integrated calibration index (ICI) and time dependant area under the receiver operator curve (tdAUC), respectively.

**Results:**

In total, 3015 patients were included. PREDICT v2.2 consistently overestimated both all-cause mortality and BCSM at 5- (ICI _all-cause mortality_ = 5.10%, 95% confidence interval (CI) 4.93–5.27; ICI_BCSM_ = 4.91%, 95% CI 4.71–5.13) and 10- years (ICI _all-cause mortality_ = 9.75%, 95% CI 9.02%–10.5, ICI_BCSM_ = 9.71%, 95% CI 8.88–10.4). In contrast, PREDICT v3.0 showed much better calibration (5 years; ICI _all-cause mortality_ = 0.69%, 95% CI 0.60–0.78; ICI_BCSM_ = 0.65%, 95% CI 0.57– 0.73; 10 years; ICI _all-cause mortality_ = 5.10%, 95% CI 4.65–5.5%; ICI_BCSM_ = 4.76%, 95% CI 4.31–5.19), although miscalibration remained in high-risk groups. Discrimination was excellent for predicting 5-year outcomes in both models but declined for 10-year predictions.

**Conclusions:**

PREDICT showed good discrimination in young patients. Although version 3.0 showed better calibration than v2.2, risk overestimation in high-risk patients may affect treatment decision-making and warrants cautious interpretation.

**Supplementary Information:**

The online version contains supplementary material available at 10.1186/s13058-026-02271-2.

## Background

Young (≤ 40 years) patients constitute 5% of newly diagnosed invasive early breast cancer in Sweden [1]. Beyond the classical clinicopathologic prognostic factors in early breast cancer, age is independently associated with inferior outcome [2–5]. This association between younger age and worse prognosis is attributed to more aggressive tumor biology, which is also reflected in gene expression and tumor phenotype levels [6, 7].

Current treatment guidelines for young patients with early breast cancer stipulate that age in addition to biological tumor factors, should guide treatment decision making [8, 9]. Gene expression profiling has been explored to aid decision on chemotherapy; however, the benefit of adjuvant chemotherapy in younger patients cannot be dismissed, even in those with low genomic risk [10, 11]. Accordingly, prognostic tools based on easily accessible patient- and tumor-related characteristics are essential for supporting clinical decisions.

Initially developed in 2010, PREDICT is the most widely used prognostic tool for supporting treatment decisions in breast cancer patients [12]. Several updates have been released, including refitted versions designed to better account for the increased risk in younger patients, as only 7% of the patients in the original cohort were younger than 40 years old [12–17]. While PREDICT performs reasonably well in validated cohorts of middle-aged patients, its prognostic estimates are less reliable for the youngest and oldest patients [13, 14, 18]. Notably, PREDICT´s performance in younger women is risk-dependent and tends to overestimate mortality in poor prognosis subgroups [13, 14]. Recently, a new version of PREDICT (v3.0) was launched, incorporating the relative harms of radiotherapy and chemotherapy [19, 20]. However, limited validation data are currently available for this version [21, 22].

The aim of the present study was to externally validate PREDICT v2.2 and v3.0 in a nationwide, population-based cohort of young (≤ 40 years) breast cancer patients in Sweden, focusing on 5- and 10-year all-cause- and breast cancer-specific mortality (BCSM) outcomes.

## Materials and methods

### Study design and main objectives

This was an analytical retrospective cohort study utilizing real-world data from a Swedish nationwide, population-based register. The objectives were to externally validate the PREDICT model (v2.2 and v3.0) using observed outcomes.

### Data source and patient selection

The National Quality Register for Breast Cancer (NKBC) in Sweden is a nationwide register containing detailed information on tumor- and treatment-related characteristics for patients diagnosed with early breast cancer with > 99% coverage using the Swedish National Cancer Register as gold standard [1]. NKBC has been used as the main data source for the research database BCBaSe 3.0 where data from NKBC (newly diagnosed patients with breast cancer between January 2008 and December 2019) has been linked to other relevant Swedish National Registries. For the scope of the current study, NKBC data has been enriched with the Cause of Death Registry, providing information on cause and date of death [23, 24].

**Fig. 1:**
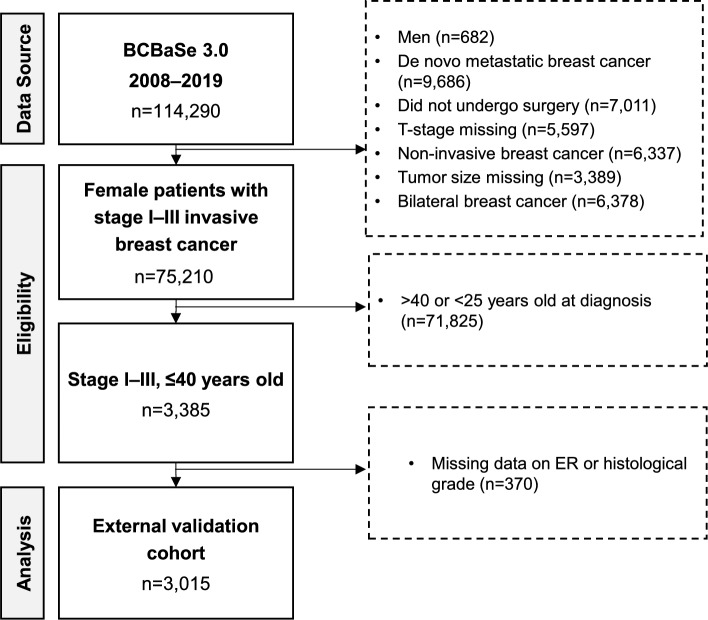
Study flowchart. *Abbreviations*: BC, breast cancer; ER, estrogen receptor

Eligible patients from the current study were patients registered in BCBaSe 3.0 who fulfilled the following inclusion criteria: female patients 25–40 years old inclusive at diagnosis, stage I–III invasive breast cancer (according to the American Joint Committee on Cancer (AJCC) 8^th^ edition [25] and treated with surgery for the primary tumor (Fig. 1). Patients with only in situ cancer were excluded from the analyses.

### Mapping of variables

The following baseline variables were included in the v2.2 model; age at diagnosis (25–40 years), postmenopausal (yes/no/unknown), estrogen receptor (ER) status (positive/negative), human epidermal growth factor receptor 2/Erb-B2 Receptor Tyrosine Kinase 2 (HER2/ERRB2) status (positive/negative/unknown), Ki67 (positive/negative/unknown (> 10% of tumor cells staining positive)), invasive tumour size (mm), tumour grade (1/2/3), detection (screening/symptoms/unknown), positive nodes (numbers), and if at least one, micrometastases only (yes/no/unknown). Additional variables in PREDICT v3.0 includes smoker (yes/never or Ex) and progesterone receptor (PR) status (positive/negative/unknown).

All PREDICT variables were available in our study cohort except for smoking habits, which has been newly introduced as a predictor for PREDICT v3.0. Accordingly, we pre-specified all patients as non-smokers (a sensitivity analysis was also performed in which all patients were defined as smokers). According to Swedish statistics, the smoking habits for this young age group should approximately correspond to on average no more than 10% during the inclusion periods, making this assumption reasonable [26]. HER2 status was defined as positive either by HER2 3 + (as assessed by immunohistochemistry) or HER2 2 + and amplification of *HER2* by in situ hybridization. For tumor grade, we used the Nottingham histological grade (NHG) system [27, 28]. ER status was defined according to Swedish national guidelines; a tumor was considered ER-positive if ≥ 10% of tumor cells stained positive and considered ER-negative if < 10% of tumor cells stained positive [27]. Patients with missing ER status and NHG data, were excluded (Fig. 1).

Regarding treatment strategies, the following treatment variables are considered in PREDICT v2.2; endocrine therapy (no/yes), chemotherapy (none, 2nd gen (standard-dose, anthracycline-based such as FEC (fluorouracil, epirubicin and cyclophosphamide))/3rd gen (high-dose, anthracycline- or taxane- based that contain taxanes such as paclitaxel and docetaxel)), trastuzumab (no/yes), bisphosphonates (no/yes). Additional treatment strategies for PREDICT v3.0 includes radiotherapy (no/yes), and if yes, the mean heart dose in Gray (Gy) is to be reported. Information on endocrine therapy (up to 5 years), radiotherapy, and chemotherapy were retrieved from NKBC. However, no data on heart dose were available and therefore, we coded 0 for right-sided and 2 for left-sided breast cancer as specified in the PREDICT instructions.

The outcomes were 5- and 10-year all-cause mortality and BCSM, defined as death due to any cause and death due to breast cancer diagnosis, respectively. For each time horizon (5 and 10 years), analyses were restricted to patients with sufficient potential follow-up, defined as having a diagnosis date at least 5 or 10 years prior to the end of registry follow-up (9 March 2021). Consequently, the number of patients included decreases for longer follow-up periods.

### Statistical analyses

We computed descriptive statistics for all variables required by PREDICT using the median and interquartile range (IQR) range for continuous variables and frequency and percentage for categorical variables. All patients retrieved from the database were entered to the PREDICT algorithms. If any variable was missing, the patient was not excluded if that variable had an “unknown” option in PREDICT. The predicted outcome for overall survival was calculated for each patient and compared with that reported in the database regarding all-cause and BCSM. The follow-up was censored at 5- and 10 years. R code containing the algorithms for PREDICT v.2.1 and 3.0 was obtained from Paul Pharoah who developed the PREDICT model (personal communication). The only difference between v2.1 and v2.2 is that the latter allows for ten years of hormone therapy, thus affecting 15-year survival (Paul Pharoah, personal communication). Since we could not validate 15-years outcomes due to insufficient follow-up, we hereafter refer to validation of v2.2.

In predictive modeling, there are two major aspects of predictive accuracy that need to be quantified: *calibration* measures the ability of the model to make unbiased estimates of the outcome, whereas *discrimination* assesses the model’s ability to separate patients’ outcomes [29]. Discrimination was assessed using the time dependent AUC (Area Under the Receiver Operating Characteristic Curve, tdAUC), accounting for competing risks (e.g. emigration, death from other causes) as required [30]. Calibration was assessed using smooth calibration curves [31]. For each prediction horizon (5- and 10- year) and outcome (breast cancer death, all-cause death), we used generalized additive models (GAM’s) to model the event status (0 = censored, 1 = event) assuming the Poisson distribution with a logarithmic link function (piece-wise exponential model) [32]. Cubic smoothing splines were used to flexibly model the baseline hazard and the complementary log–log of predicted risk (that computed by PREDICT). We relaxed the proportional hazards assumption by including a tensor product interaction term between the above terms, but with an additional shrinkage penalty allowing the term to shrink towards zero. For the competing risk cases (e.g. emigration, death from other cause), cause was modelled as a categorical parametric term which was allowed to vary (interact) with the baseline hazard and predicted risk. As above, an additional penalty was applied to the interaction terms allowing the term to shrink towards zero. We then plotted the model-based estimate of observed risk against predicted risk with 95% confidence intervals (CIs). From the fitted model, we computed the Integrated Calibration Index (ICI), defined as the mean absolute difference between “observed” (model-based) and predicted risk. Confidence intervals for tdAUC and ICI were computed using the percentile bootstrap with 200 replications.

We assessed calibration and discrimination for the whole cohort, as well as for prognostic subgroups that reflect clinical and anatomical risks, namely, molecular subtype (Luminal (ER + /HER2 negative (HER2–)), HER2 positive (HER2 +), triple-negative breast cancer (TNBC)), as well as according to nodal status (N0; node-negative, and N + ; node-positive). Comparison between the models was performed assuming all patients as both smokers and non-smokers in the v3.0.

Statistical analysis was conducted in R [30, 33], relying heavily on the tidyverse suite, mgcv pammtools and tdROC. For differences in ICI or tdROC, CI not including zero was considered statistically significant. R code to fully reproduce the analyses may be requested from the corresponding author.

## Results

### Baseline characteristics

Descriptive statistics for baseline variables for 5- and 10-year outcomes are shown in Table 1, illustrating the total number of patients for each follow-up time period as well as in subgroups of interest. Of the 3015 patients included in the cohort, 1883 had potential follow-up for 5-year analysis and 726 for 10-year analysis, based on the registry follow-up cut-off. Between 2008 and 2019, 1883 women, with a median age at diagnosis of 37 years (IQR = 34–39) were eligible for the 5-year follow-up analyses. In total, 107 (5.7%) patients died from breast cancer whilst 8 (0.4%) and 21 (1.1%) died from other causes or emigrated, respectively. Most women (based on 5 years follow-up cohort, n = 1883)) had tumors that were ER-positive (n = 1387; 74%), PR-positive (n = 1235; 66%), HER2-negative (n = 1448; 77%) and histological grade 3 (n = 1031; 55%). More patients were node-negative (N0; n = 1135; 60%). Overall, approximately half of the cohort received endocrine therapy (n = 1059; 56%) and in total 769 (41%) and 345 (18%), received standard anthracycline and taxane/high-dose anthracycline chemotherapy, respectively. The corresponding values for patients (n = 726) in the 10-year follow-up cohort are presented in Table 1.

**Table 1:** Patient- and tumor characteristics for all patients, and for subgroups of interest at 5 and 10 years follow-up, respectively

Variable	All		Node-negative		Node-positive		Triple-negative		Luminal		HER2 +	
	5y n = 1883^1^	10y n = 726^1^	5y n = 1135^1^	10y n = 418^1^	5y n = 748^1^	10y n = 308^1^	5y n = 353^1^	10y n = 145^1^	5y n = 1095^1^	10y n = 395^1^	5y n = 390^1^	10y n = 155^1^
**Events**												
Censored	1747 (93%)	611 (84%)	1080 (95%)	369 (88%)	667 (89%)	242 (79%)	302 (86%)	120 (83%)	1043 (95%)	334 (85%)	363 (93%)	133 (86%)
Died from breast cancer	107 (5.7%)	94 (13%)	37 (3.3%)	37 (8.9%)	70 (9.4%)	57 (19%)	47 (13%)	25 (17%)	38 (3.5%)	48 (12%)	16 (4.1%)	14 (9.0%)
Died from other causes	8 (0.4%)	12 (1.7%)	6 (0.5%)	6 (1.4%)	2 (0.3%)	6 (1.9%)	3 (0.8%)		3 (0.3%)	9 (2.3%)	2 (0.5%)	3 (1.9%)
Emigrated	21 (1.1%)	9 (1.2%)	12 (1.1%)	6 (1.4%)	9 (1.2%)	3 (1.0%)	1 (0.3%)		11 (1.0%)	4 (1.0%)	9 (2.3%)	5 (3.2%)
Age at diagnosis (years)	37 (34, 39)	37 (34, 39)	37 (34, 39)	37 (34, 39)	37 (34, 39)	37 (34, 39)	36 (32, 38)	35 (32, 38)	38 (35, 40)	38 (35, 40)	37 (33, 39)	37 (34, 39)
**ER**												
Negative	496 (26%)	203 (28%)	312 (27%)	127 (30%)	184 (25%)	76 (25%)	353 (100%)	145 (100%)	19 (1.7%)	5 (1.3%)	110 (28%)	42 (27%)
Positive	1387 (74%)	523 (72%)	823 (73%)	291 (70%)	564 (75%)	232 (75%)			1076 (98%)	390 (99%)	280 (72%)	113 (73%)
**PR**												
Negative	644 (34%)	274 (38%)	398 (35%)	167 (40%)	246 (33%)	107 (35%)	353 (100%)	145 (100%)	120 (11%)	52 (13%)	153 (39%)	64 (41%)
Positive	1235 (66%)	450 (62%)	734 (65%)	250 (60%)	501 (67%)	200 (65%)			975 (89%)	343 (87%)	237 (61%)	91 (59%)
Unknown	4 (0.2%)	2 (0.3%)	3 (0.3%)	1 (0.2%)	1 (0.1%)	1 (0.3%)						
**HER2**												
Negative	1448 (77%)	540 (74%)	898 (79%)	320 (77%)	550 (74%)	220 (71%)	353 (100%)	145 (100%)	1,095 (100%)	395 (100%)		
Positive	392 (21%)	156 (21%)	212 (19%)	82 (20%)	180 (24%)	74 (24%)					390 (100%)	155 (100%)
Unknown	43 (2.3%)	30 (4.1%)	25 (2.2%)	16 (3.8%)	18 (2.4%)	14 (4.5%)						
**Ki67**^**2**^												
Negative	96 (5.1%)	20 (2.8%)	76 (6.7%)	14 (3.3%)	20 (2.7%)	6 (1.9%)	1 (0.3%)		91 (8.3%)	20 (5.1%)	3 (0.8%)	
Positive	865 (46%)	81 (11%)	528 (47%)	53 (13%)	337 (45%)	28 (9.1%)	166 (47%)	22 (15%)	496 (45%)	38 (9.6%)	196 (50%)	19 (12%)
Unknown	922 (49%)	625 (86%)	531 (47%)	351 (84%)	391 (52%)	274 (89%)	186 (53%)	123 (85%)	508 (46%)	337 (85%)	191 (49%)	136 (88%)
Tumor size (mm)	20 (13, 28)	20 (14, 30)	17 (12, 24)	18 (12, 25)	23 (17, 35)	25 (18, 35)	22 (16, 31)	22 (16, 30)	18 (12, 27)	19 (13, 28)	20 (14, 29)	22 (15, 29)
**Tumour grade**												
1	201 (11%)	79 (11%)	160 (14%)	63 (15%)	41 (5.5%)	16 (5.2%)	2 (0.6%)	2 (1.4%)	187 (17%)	72 (18%)	9 (2.3%)	4 (2.6%)
2	651 (35%)	232 (32%)	394 (35%)	128 (31%)	257 (34%)	104 (34%)	13 (3.7%)	6 (4.1%)	513 (47%)	177 (45%)	111 (28%)	42 (27%)
3	1031 (55%)	415 (57%)	581 (51%)	227 (54%)	450 (60%)	188 (61%)	338 (96%)	137 (94%)	395 (36%)	146 (37%)	270 (69%)	109 (70%)
**Detection mode**												
Clinically detected	1610 (86%)	627 (86%)	962 (85%)	357 (85%)	648 (87%)	270 (88%)	328 (93%)	137 (94%)	895 (82%)	323 (82%)	347 (89%)	138 (89%)
Screen detected	266 (14%)	95 (13%)	169 (15%)	58 (14%)	97 (13%)	37 (12%)	24 (6.8%)	7 (4.8%)	196 (18%)	70 (18%)	41 (11%)	16 (10%)
Unknown	7 (0.4%)	4 (0.6%)	4 (0.4%)	3 (0.7%)	3 (0.4%)	1 (0.3%)	1 (0.3%)	1 (0.7%)	4 (0.4%)	2 (0.5%)	2 (0.5%)	1 (0.6%)
**Number positive lymph nodes**												
0	1135 (60%)	418 (58%)	1,135 (100%)	418 (100%)			232 (66%)	95 (66%)	666 (61%)	225 (57%)	211 (54%)	82 (53%)
1	317 (17%)	130 (18%)			317 (42%)	130 (42%)	57 (16%)	24 (17%)	175 (16%)	75 (19%)	74 (19%)	24 (15%)
2	152 (8.1%)	57 (7.9%)			152 (20%)	57 (19%)	29 (8.2%)	12 (8.3%)	96 (8.8%)	31 (7.8%)	26 (6.7%)	13 (8.4%)
3	86 (4.6%)	39 (5.4%)			86 (11%)	39 (13%)	12 (3.4%)	6 (4.1%)	53 (4.8%)	25 (6.3%)	20 (5.1%)	7 (4.5%)
4	43 (2.3%)	20 (2.8%)			43 (5.7%)	20 (6.5%)	4 (1.1%)	1 (0.7%)	26 (2.4%)	10 (2.5%)	11 (2.8%)	7 (4.5%)
≥ 5	150 (8.0%)	62 (8.5%)			150 (20%)	62 (20%)	19 (5.4%)	7 (4.8%)	79 (7.2%)	29 (7.3%)	48 (12%)	22 (14%)
**Radiotherapy**												
No	809 (43%)	466 (64%)	580 (51%)	298 (71%)	229 (31%)	168 (55%)	163 (46%)	103 (71%)	442 (40%)	246 (62%)	178 (46%)	97 (63%)
Yes	1074 (57%)	260 (36%)	555 (49%)	120 (29%)	519 (69%)	140 (45%)	190 (54%)	42 (29%)	653 (60%)	149 (38%)	212 (54%)	58 (37%)
**Radiation to heart (grays)**												
0	1298 (69%)	589 (81%)	816 (72%)	350 (84%)	482 (64%)	239 (78%)	248 (70%)	121 (83%)	743 (68%)	320 (81%)	277 (71%)	125 (81%)
2	585 (31%)	137 (19%)	319 (28%)	68 (16%)	266 (36%)	69 (22%)	105 (30%)	24 (17%)	352 (32%)	75 (19%)	113 (29%)	30 (19%)
**Endocrine therapy**												
No	824 (44%)	465 (64%)	517 (46%)	283 (68%)	307 (41%)	182 (59%)	341 (97%)	143 (99%)	293 (27%)	216 (55%)	169 (43%)	89 (57%)
Yes	1059 (56%)	261 (36%)	618 (54%)	135 (32%)	441 (59%)	126 (41%)	12 (3.4%)	2 (1.4%)	802 (73%)	179 (45%)	221 (57%)	66 (43%)
**Chemotherapy**												
None	769 (41%)	475 (65%)	541 (48%)	295 (71%)	228 (30%)	180 (58%)	114 (32%)	90 (62%)	523 (48%)	281 (71%)	107 (27%)	83 (54%)
Standard anthracycline	769 (41%)	238 (33%)	415 (37%)	116 (28%)	354 (47%)	122 (40%)	165 (47%)	50 (34%)	391 (36%)	110 (28%)	197 (51%)	68 (44%)
Taxanes / high-dose anthracycline	345 (18%)	13 (1.8%)	179 (16%)	7 (1.7%)	166 (22%)	6 (1.9%)	74 (21%)	5 (3.4%)	181 (17%)	4 (1.0%)	86 (22%)	4 (2.6%)
**Trastuzumab therapy**												
No	1582 (84%)	641 (88%)	975 (86%)	376 (90%)	607 (81%)	265 (86%)	351 (99%)	145 (100%)	1,089 (99%)	393 (99%)	100 (26%)	74 (48%)
Yes	301 (16%)	85 (12%)	160 (14%)	42 (10%)	141 (19%)	43 (14%)	2 (0.6%)		6 (0.5%)	2 (0.5%)	290 (74%)	81 (52%)
**Bisphosphonate therapy**												
No	1872 (99%)	726 (100%)	1132 (100%)	418 (100%)	740 (99%)	308 (100%)	353 (100%)	145 (100%)	1087 (99%)	395 (100%)	387 (99%)	155 (100%)
Yes	11 (0.6%)		3 (0.3%)		8 (1.1%)				8 (0.7%)		3 (0.8%)	

^1^n (%); ^2^ positive defined as > 10% according to the PREDICT algorithm; Median (Q1, Q3)

*Abbreviations*: ER, Estrogen receptor; HER2, Human epidermal growth factor receptor; PR, Progesterone receptor

### Performance of PREDICT v2.2 vs v3.0 in all patients at 5- and 10 years follow-up

**Fig. 2:**
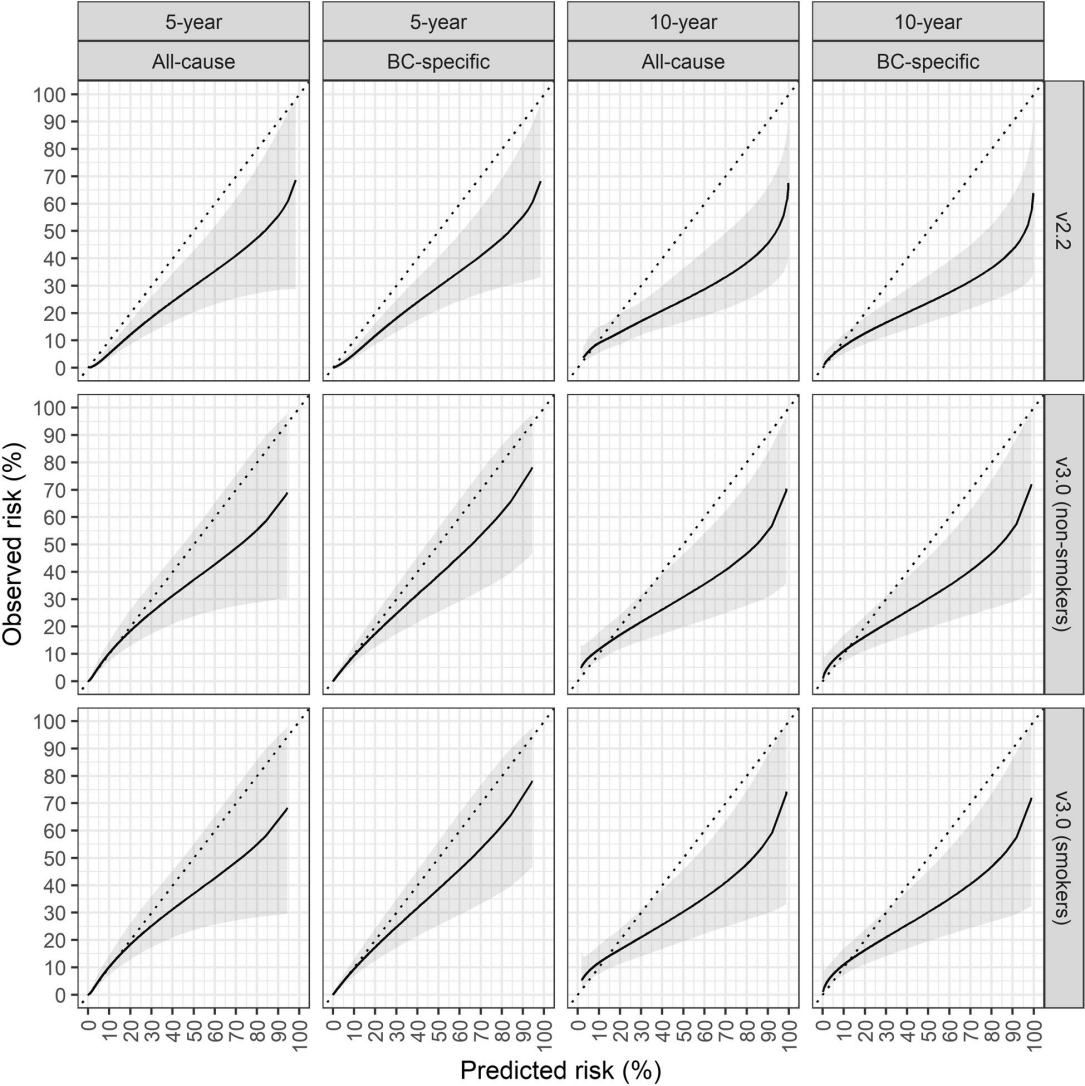
Calibration plots for PREDICT v2.2 and v3.0. Observed and predicted risks (%) for all patients with respect to both all-cause and breast cancer-specific survival. V3.0 is illustrated assuming all patients to be smokers or non-smokers. *Abbreviations*: BC, breast cancer

**Fig. 3:**
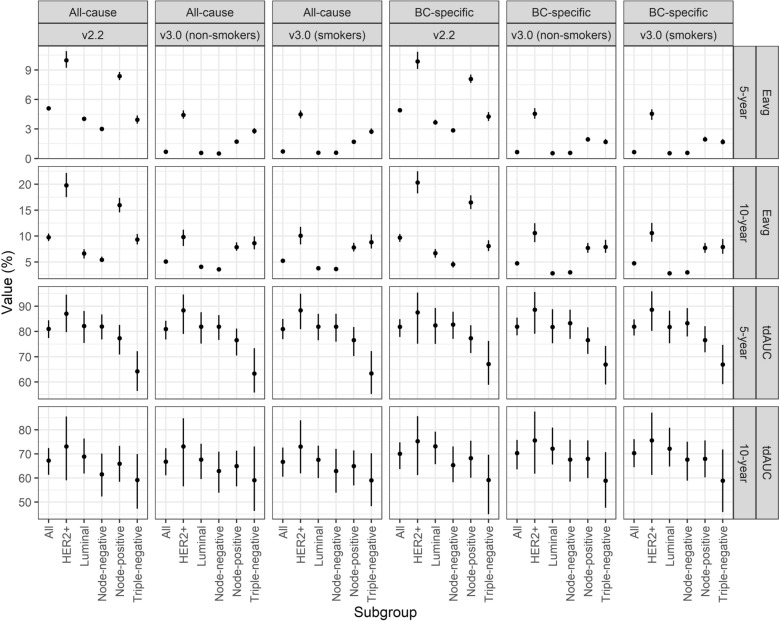
Calibration and discriminatory values (%) for all patients and specific subgroups. Calibration (Eavg) and discriminatory (tdAUC) values for all patients and in subgroups of interest with respect to both all-cause and breast cancer-specific survival. V3.0 is illustrated assuming all patients to be smokers or non-smokers. *Abbreviations*: BC, breast cancer; HER2, human epidermal growth factor receptor; tdAUC, time dependent area under the receiver operating characteristic curve

**Fig. 4:**
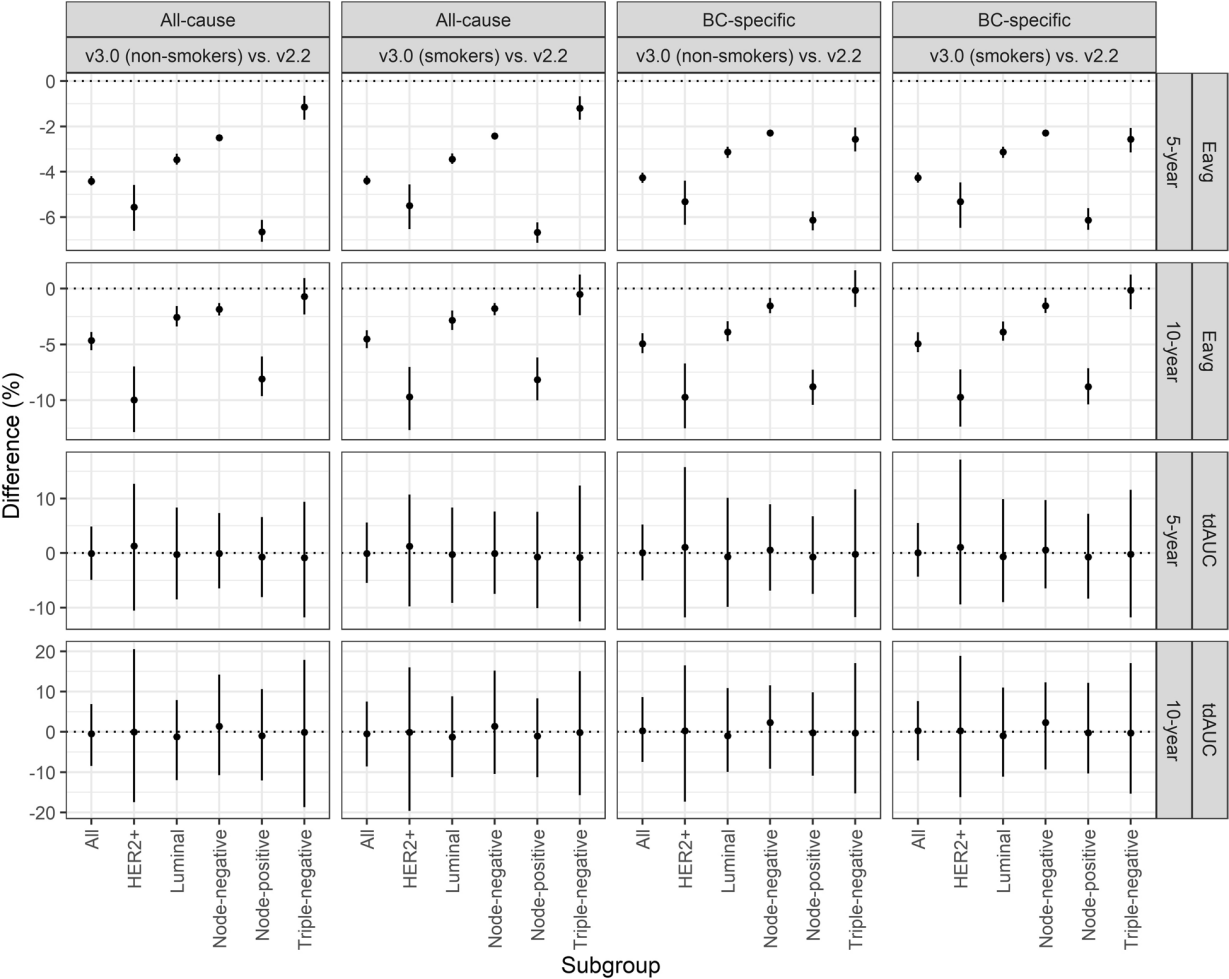
Calibration and discriminatory differences (%) for all patients and specific subgroups. Calibration (Eavg) and discriminatory (tdAUC) differences for all patients and in subgroups of interest with respect to both all-cause and breast cancer-specific survival. V3.0 is illustrated assuming all patients to be smokers or non-smokers. *Abbreviations*: BC, breast cancer; HER2, human epidermal growth factor receptor; tdAUC, time dependent area under the receiver operating characteristic curve

Calibration curves revealed excellent performance of PREDICT at low risk of death (between approximately 0–20% probability of death), though the calibration curve departed from the line of unity at higher risk (approximately 20–80%) but tended back towards the line of unity at very high risk (Fig. 2). This was true for both model v2.2 and v3.0, and no difference was noted regarding smoking status. PREDICT v2.2 overestimated risk of death at 5 years (ICI_all-cause mortality_ = 5.10%, 95% CI 4.93–5.27; ICI_BCSM_ (ICI = 4.91%, 95% CI 4.71–5.13), and the magnitude of overestimation increased at 10 years (ICI_all-cause mortality_ = 9.75%, 95% CI 9.02–10.5; ICI_BCSM_ = 9.71%, 95% CI 8.88–10.4) (Table 2 and Fig. 3). PREDICT v3.0 was better calibrated than v2.2 at both time horizons, though again performed better at 5 years (ICI_all-cause mortality_ = 0.69%, 95% CI 0.66–0.78; ICI_BCSM_ = 0.65%, 95% CI 0.57–0.73) compared to 10 years (ICI_all-cause mortality_ = 5.10%, 95% CI 4.65–5.52; ICI_BCSM_ = 4.76%, 95% CI 4.31–5.19) (Table 2 and Figs. 3–4). The difference in performance was significantly better for v3.0 as compared to v2.2 for both outcomes and time horizons (Table 2 and Fig. 4).

**Table 2:** Calibration and discrimination of PREDICT v2.2 and v3.0 in all patients and in different subgroup across 5- and 10 years horizons

Horizon	Death	Integrated calibration index (%)			Time dependent AUC (%)		
		v.3.0	v.2.2	v.3.0-v2.2^a^	v.3.0	v.2.2	v.3.0-v2.2^a^
**All patients**							
5-year	All-cause	0.69 [0.60, 0.78]	5.10 [4.93, 5.27]	− 4.42 [− 4.61, − 4.20]	81.01 [77.38, 84.43]	80.95 [76.82, 84.19]	− 0.06 [− 4.91, 4.85]
	BC-specific	0.65 [0.57, 0.73]	4.91 [4.71, 5.13]	− 4.26 [− 4.49, − 4.04]	81.83 [77.81, 84.83]	81.92 [78.44, 85.47]	0.09 [− 5.01, 5.25]
10-year	All-cause	5.10 [4.65, 5.52]	9.75 [9.02, 10.49]	− 4.66 [− 5.53, − 3.88]	67.21 [61.29, 72.46]	66.76 [61.11, 72.33]	− 0.46 [− 8.43, 6.92]
	BC-specific	4.76 [4.31, 5.19]	9.71 [8.88, 10.42]	− 4.95 [− 5.79, − 4.00]	70.09 [63.67, 74.80]	70.33 [63.55, 75.79]	0.25 [− 7.50, 8.68]
**HER2 + **							
5-year	All-cause	4.42 [4.04, 4.88]	9.98 [9.21, 10.91]	− 5.56 [− 6.60, − 4.58]	87.05 [79.72, 94.57]	88.34 [78.99, 94.58]	1.28 [− 10.55, 12.71]
	BC-specific	4.56 [4.04, 5.11]	9.87 [9.09, 10.84]	− 5.31 [− 6.34, − 4.39]	87.54 [75.12, 95.36]	88.61 [79.07, 95.64]	1.07 [− 11.79, 15.78]
10-year	All-cause	9.81 [8.08, 11.22]	19.78 [17.50, 22.18]	− 9.97 [− 12.86, − 6.99]	73.08 [59.01, 85.53]	73.06 [56.51, 84.75]	− 0.02 [− 17.43, 20.57]
	BC-specific	10.59 [8.82, 12.49]	20.34 [18.24, 22.50]	− 9.74 [− 12.54, − 6.70]	75.28 [61.17, 85.66]	75.58 [61.76, 87.56]	0.30 [− 17.31, 16.49]
**Luminal**							
5-year	All-cause	0.57 [0.49, 0.67]	4.04 [3.80, 4.24]	− 3.47 [− 3.69, − 3.20]	82.12 [75.40, 88.12]	81.88 [75.18, 87.58]	− 0.23 [− 8.50, 8.35]
	BC-specific	0.54 [0.48, 0.60]	3.67 [3.44, 3.94]	− 3.13 [− 3.39, − 2.90]	82.42 [75.07, 89.29]	81.76 [75.29, 88.80]	− 0.66 [− 9.87, 10.13]
10-year	All-cause	4.07 [3.92, 4.25]	6.63 [5.65, 7.47]	− 2.56 [− 3.40, − 1.57]	68.86 [61.87, 76.39]	67.64 [59.51, 74.18]	− 1.22 [− 12.01, 7.93]
	BC-specific	2.82 [2.71, 2.95]	6.71 [5.83, 7.51]	− 3.89 [− 4.72, − 2.93]	73.11 [65.75, 79.21]	72.18 [65.62, 80.90]	− 0.94 [− 9.97, 10.88]
**Triple-negative**							
5-year	All-cause	2.79 [2.51, 3.09]	3.94 [3.54, 4.37]	− 1.15 [− 1.71, − 0.66]	64.19 [56.42, 72.16]	63.32 [55.84, 73.36]	− 0.87 [− 11.77, 9.43]
	BC-specific	1.68 [1.45, 1.98]	4.25 [3.81, 4.71]	− 2.57 [− 3.10, − 2.05]	67.09 [58.89, 76.23]	66.89 [58.99, 74.23]	− 0.20 [− 11.74, 11.67]
10-year	All-cause	8.63 [7.42, 9.93]	9.33 [8.39, 10.39]	− 0.70 [− 2.33, 0.95]	59.17 [47.24, 69.95]	59.07 [46.32, 73.05]	− 0.10 [− 18.70, 17.91]
	BC-specific	7.91 [6.76, 9.26]	8.06 [7.11, 9.19]	− 0.16 [− 1.65, 1.64]	59.12 [44.99, 69.69]	58.87 [47.64, 70.73]	− 0.25 [− 15.30, 17.05]
**Node-negative**							
5-year	All-cause	0.51 [0.49, 0.53]	3.01 [2.95, 3.07]	− 2.50 [− 2.57, − 2.43]	81.96 [76.81, 86.72]	81.87 [76.65, 86.46]	− 0.09 [− 6.47, 7.35]
	BC-specific	0.56 [0.55, 0.58]	2.86 [2.78, 2.96]	− 2.29 [− 2.39, − 2.21]	82.71 [77.09, 87.78]	83.27 [77.05, 88.57]	0.55 [− 6.90, 8.93]
10-year	All-cause	3.59 [3.32, 3.84]	5.42 [5.00, 6.02]	− 1.84 [− 2.40, − 1.30]	61.54 [52.30, 70.09]	62.92 [53.86, 70.90]	1.38 [− 10.78, 14.23]
	BC-specific	3.02 [2.75, 3.28]	4.54 [3.91, 5.13]	− 1.53 [− 2.22, − 0.84]	65.30 [58.24, 73.10]	67.65 [58.50, 75.85]	2.35 [− 9.14, 11.58]
**Node-positive**							
5-year	All-cause	1.72 [1.56, 1.93]	8.37 [7.96, 8.79]	− 6.65 [− 7.09, − 6.12]	77.29 [70.86, 82.57]	76.59 [70.48, 81.13]	− 0.70 [− 8.07, 6.61]
	BC-specific	1.94 [1.75, 2.19]	8.07 [7.67, 8.53]	− 6.13 [− 6.58, − 5.75]	77.29 [71.44, 82.45]	76.58 [71.13, 81.63]	− 0.71 [− 7.51, 6.77]
10-year	All-cause	7.86 [7.15, 8.78]	15.95 [14.56, 17.37]	− 8.09 [− 9.64, − 6.08]	65.91 [58.43, 73.32]	64.92 [56.50, 71.32]	− 0.99 [− 12.08, 10.66]
	BC-specific	7.71 [6.77, 8.66]	16.50 [15.21, 17.86]	− 8.79 [− 10.43, − 7.27]	68.19 [60.08, 75.47]	67.97 [59.95, 75.59]	− 0.22 [− 10.88, 9.84]

^a^difference between PREDICT versions

Assumed non-smokers for PREDICT v3.0

*Abbreviations*: AUC, Area under curve; BC, Breast cancer; HER2, Human epidermal growth factor receptor; v., Version of PREDICT

Discrimination was excellent for 5-year risks and was similar across outcomes and PREDICT versions (v2.2: tdAUC_all-cause mortality_ = 80.1%, 95% CI 76.8–84.2; v2.2: tdAUC_BCSM_ = 81.9%, 95% CI 78.4–85.5; v3.0: tdAUC_all-cause mortality_ = 81.0%, 95% CI 77.4–84.4; v3.0: tdAUC_BCSM_ = 81.8%, 95% CI 77.8–84.8 (Table 2, Figs. 3 and 4). Discriminative ability decreased for 10-year risk, though was again similar between PREDICT versions and outcomes (v2.2: tdAUC_all-cause mortality_ = 66.8%, 95% CI 61.1–72.3; v2.2: tdAUC_BCSM_ = 70.3%, 95% CI 63.6–75.8; v3.0: tdAUC_all-cause mortality_ = 67.2%, 95% CI 61.3–72.5; v3.0: tdAUC_BCSM_ = 70.1%, 95% CI 63.7–74.8).

### Performance of PREDICT v2.2 vs v3.0 by molecular subtype and nodal status

Subgroup analyses by molecular subtype and nodal status (Tables 2) showed substantial overestimation by PREDICT v2.2 in HER2-positive patients, with ~ 10% miscalibration at 5 years (ICI_all-cause mortality_ = 10.0%, 95% CI 9.21–10.9, ICI_BCSM_ = 9.9%, 95% CI 9.09–10.8), and ~ 20% at 10 years. In contrast, v3.0 reduced miscalibration to ~ 4.5% at 5 years (ICI_all-cause mortality_ = 4.42%, 95% CI 4.04–4.88; ICI_BCSM_ = 4.56%, 95% CI 4.04–5.11) and ~ 10% at 10 years (Table 2 and Figs. 3–4). In TNBC, both versions showed similar moderate overestimation, with 2–4% error at 5 years and 8–9% at 10 years. In node-negative cases, both models showed good accuracy, with minimal overestimation by PREDICT v3.0 (≤ 0.5%) at 5 years. In node-positive disease, overestimation was substantial, particularly for v2.2. At 10 years, ICIs reached 16.5% (95% CI 15.2–17.9) and 7.7% (95% CI 6.77– 8.66) for PREDICT v2.2 and v3.0, respectively. All data are detailed in Table 2. Statistical differences between the models were seen across all subgroups, except for TNBC at 10 years for both outcomes (Table 2 and Fig. 4). Calibration plots for the different subgroups are available as Supplementary files (Supplementary Figure S1–5).

Discriminatory performance across subgroups is summarized in Table 2 and Figs. 3–4). Notably, reduced discriminatory capacity was observed in patients with TNBC at 10 years, with tdAUCs for all-cause mortality of 59.1% (95% CI 46.3–73.1) for PREDICT v2.2 and 59.2% (95% CI 47.2–70.0) for v3.0. Similar patterns were observed for BCSM, and no significant differences were noted between the two PREDICT models.

## Discussion

This external validation of the prognostic model PREDICT was conducted using real-world data from a large, contemporary Swedish cohort of breast cancer patients aged ≤ 40 years at diagnosis. Our analysis showed that PREDICT tended to overestimate both all-cause mortality and BCSM, and more pronounced at 10-year follow-up. The overestimation was particularly notable among high-risk patients, such as those with HER2-positive and node-positive disease. PREDICT version 3.0 showed improved accuracy over version 2.2 across both outcomes and time horizons.

Prior studies of older PREDICT versions have reported mixed results depending on the prognostic risk groups under study [13, 14, 34]. A Dutch validation study in patients < 50 years old found PREDICT v1.3 underestimated 10-year all-cause mortality in “good” prognosis subgroups but overestimated this endpoint in “poor” prognosis subgroups [13]. Maishman et al. observed that PREDICT v1.1 was more accurate at 8–10 years than at 5 years, but it underestimated 5-year deaths by 56% in ER-positive tumors, while overestimated deaths by 21% in ER-negative tumors [14]. Similarly, Wang et al. found that PREDICT v2.2 underestimated 10-year all-cause mortality by 33% in untreated node-negative patients < 40 years, particularly in ER-positive cases [35]. Limited data exists for PREDICT v3.0, but SEER results suggest similar miscalibration patterns by ER status (− 8% in ER-positive, + 2% in ER-negative patients) for 10-year all-cause mortality [22]. According to our data, PREDICT v3.0 demonstrated a 67–82% ability to distinguish between survivors and non-survivors across prediction horizons, however with a higher performance at 5 compared to 10 years. The results are consistent with findings from Grootes et al. [20] and a study from United States [22] validating the v3.0 model. We validated both all-cause and BCS mortality, i.e. cause of death was known. The results regarding these outcomes were in general similar, and not surprising since young patients are not expected to have competing risk of deaths other than breast cancer. Our calibration data indicate that PREDICT v3.0 overestimated 5-year all-cause and BCS mortality, but only on average 0.7%. The figures for v2.2 were less accurate, indicating an overestimation of on average 5% and 10% after 5- and 10 years follow-up, respectively. In general, a more pronounced miscalibration was detected in high-risk patients, also apparent in prespecified subgroup analyses. Our findings demonstrated a clear improvement in predictive performance with PREDICT v3.0, compared to v2.2, also reported in the recent publication from the U.S [22]. This suggests better calibration in younger patients and may reflect improved risk–benefit alignment in estimating treatment effects.

The clinical relevance of PREDICT’s tendency to overestimate mortality, particularly in version 2.2, is nevertheless substantial, as it may significantly influence adjuvant treatment decisions in young patients with breast cancer, especially those classified as high-risk. Assuming a threshold for clinical benefit from adjuvant systemic therapy of ≥ 3%, even minor degrees of model miscalibration could have a direct impact on therapeutic decision-making. While calibration appeared acceptable for low-risk patients, the extent of miscalibration increased substantially among high-risk individuals. For these patients, especially over a 10-year horizon, PREDICT v2.2 overestimated mortality by as much as 30 to 40 percentage points. Given the longer life expectancy and reduced influence of competing mortality risks in younger patients, precise prognostic stratification in this population is imperative. Overestimation of absolute treatment benefit in poor-prognosis subgroups may lead to overtreatment, as the perceived benefit may be overrepresented. This was less pronounced in v3.0 by our results. The authors behind this updated version of PREDICT, concluded that the v3.0 re-classified patients, in that 38% of those considered as high risk (by Cambridge Unit) by v2.2 would be low according to v3.0 and spared the harms of treatment [20].

The more pronounced miscalibration, particularly observed in high-risk groups, likely reflects a combination of factors related to both model structure and evolving clinical practice. First, there were few young patients included in the PREDICT development cohort (2% < 35 years old) and second, the inclusion period in that cohort was 1999–2003 [36], and this differs by a decade as compared to our cohort. Third, inaccurate estimation of treatment benefits to newer treatment strategies could explain the miscalibration of contemporary patients, since the relative risk reduction of treatment in PREDICT are derived from the Early Breast Cancer Trialists’ Collaborative Group (EBCTCG*)* meta-analyses [37, 38]. Patients classified as high-risk typically have greater exposure to systemic therapies and more aggressive disease biology, and therefore benefit most from advances in treatment that are not fully captured in earlier model versions. As a result, absolute risks may be overestimated when historical treatment effects are applied to contemporary patients. In addition, geographical differences in clinical practice, population characteristics, and subtype distribution between the development cohorts and our Swedish population may also contribute to the observed miscalibration. Finally, biological features of tumors in young patients that cannot be captured with the current clinicopathological factors, makes extrapolation from prediction models developed mainly from older populations uncertain. In addition, small inaccuracies in relative risk estimates or baseline risk can become amplified at higher predicted risk levels, leading to greater absolute miscalibration in these groups. Notably, this pattern was substantially attenuated in PREDICT v3.0, suggesting that incorporation of more recent treatment effects improves calibration, although some overestimation remains.

PREDICT exhibited very good- to excellent- discriminatory performance across the cohort. As discrimination is determined by the coefficients from the underlying cause-specific Cox proportional hazards models, recalibration using population-specific coefficients is unlikely to yield major gains. Instead, the observed miscalibration likely originates from inaccuracies in the baseline hazard functions, suggesting that recalibrating these baselines could further improve model performance. Given the representativeness of our cohort in terms of patient and tumor characteristics for the broader young breast cancer population, our findings are likely generalizable. However, evolving therapeutic standards and the rapid incorporation of novel agents underscore the need for continuous updates of prognostic tools. Future efforts should aim to enhance model validity by integrating additional prognostic factors including genomic assays and expanding follow-up durations. Moreover, subsequent versions of PREDICT may benefit from continued recalibration to contemporary populations and from incorporating more detailed treatment information, particularly for patients at the highest risk as discussed above. One could also discuss the value of adding more unmeasurable variables such as socioeconomic status and comorbidities, however, one must also keep in mind that the PREDICT model should be user-friendly and the variables easy to categorise.

This study has several strengths, including a large, population-based cohort and high-quality data with accurate cause-of-death classification. It represents an independent validation using robust methodology. Limitations include incomplete data for some PREDICT v3.0 variables, notably smoking status, and cardiac radiotherapy dose. As smoking was a key missing variable, all patients were assumed non-smokers and assumed as reasonable based on national statistics for this age group [26], which was also supported by sensitivity analyses illustrating similar results. As presented in Table 1, there was a certain proportion of patients that did not receive standard treatment (anti-HER2 in the HER2 positive and endocrine therapy in the Luminal subgroup, respectively) and these data should affect both endpoints at both time horizons. Nevertheless, the PREDICT overestimated mortality in these subgroups. Lastly, we were unable to estimate outcomes beyond 10 years, which may limit relevance for younger patients.

## Conclusions

Recent and previous PREDICT models are generally accurate for outcome estimation in breast cancer patients ≤ 40 years old but tend to overestimate risk in high-risk subgroups, particularly at 10 years. While the updated version shows improvement, it should be used cautiously in young high-risk patients, as it may overestimate risks and impact treatment decisions.

## Supplementary Information

Supplementary Material 1.

## Data Availability

All data are available upon reasonable request.

## Abbreviations

AJCC: American joint committee on cancer
BCSM: Breast cancer-specific mortality
CI: Confidence interval
ER: Estrogen receptor
ERRB2: Erb-B2 receptor tyrosine kinase 2
GAM: Generalized additive model
Gy: Gray
ICI: Integrated calibration index
IQR: Interquartile range
HER2: Human epidermal growth factor receptor 2
NHG: Nottingham histological grade
NKBC: The swedish national quality register for breast cancer
PR: Progesterone receptor
tdAUC: Time dependent area under the receiver operating characteristic curve
TNBC: Triple-negative breast cancer
