# Supplementary material for: PREDICT v3.0 outperforms v2.2 in young (25–40 years) breast cancer patients according to real-world data from a large Swedish population-based registry
Source: Breast Cancer Res. 2026 Mar 31;28:70. doi: 10.1186/s13058-026-02271-2 (PMC13064026; doi:10.1186/s13058-026-02271-2)
Supplement: Supplementary file 1 — Supplementary Material 1. [file 13058_2026_2271_MOESM1_ESM.docx]

**SUPPLEMENTARY FILES**


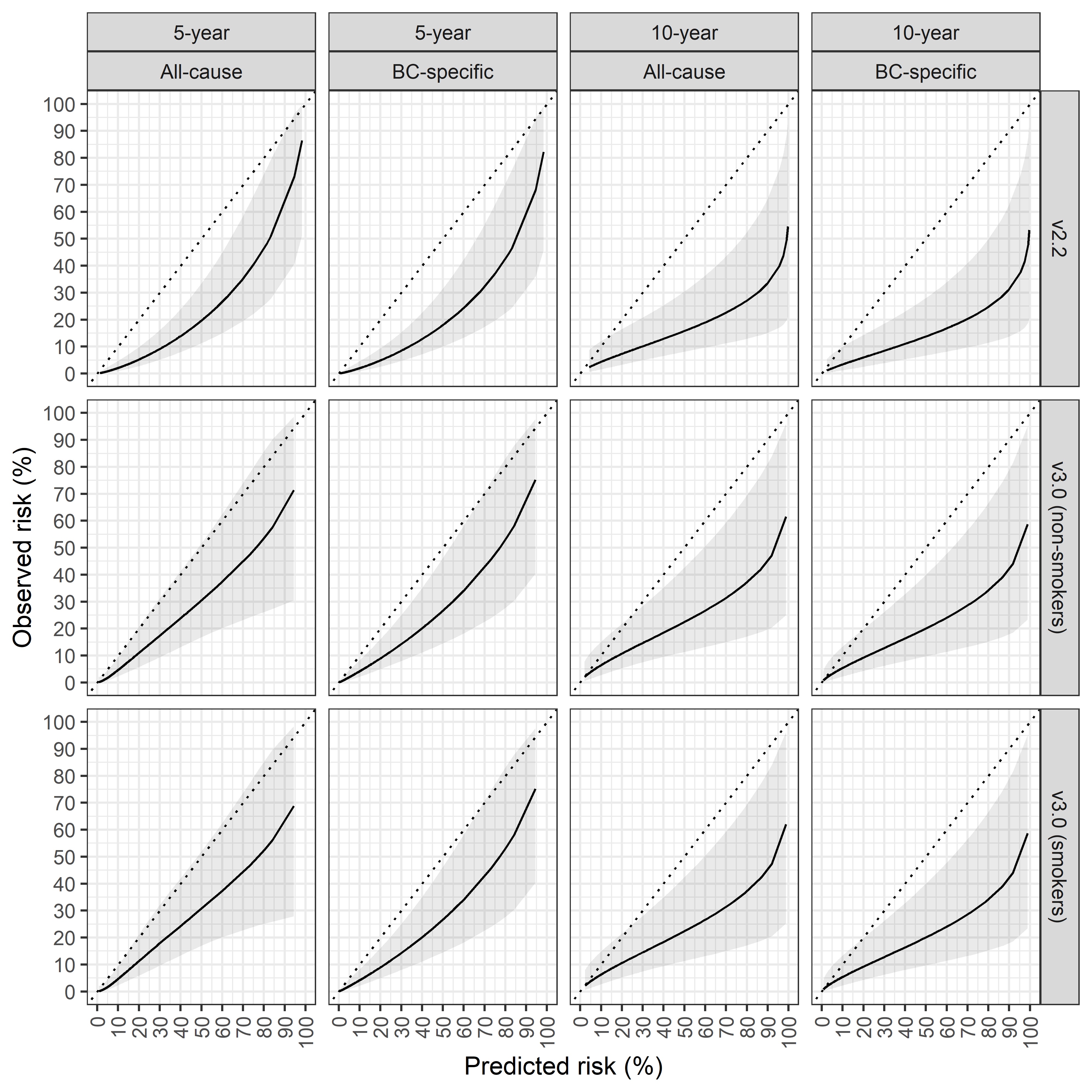


**Figure S1.** Calibration plots for PREDICT v2.2 and v3.0 in HER2-positive patients

Observed and predicted risks (%) for patients with HER2-positive tumors with respect to both all-cause and breast cancer-specific survival. V3.0 is illustrated assuming all patients to be smokers or non-smokers.

Abbreviations: BC, breast cancer; HER2, human epidermal growth factor receptor 2


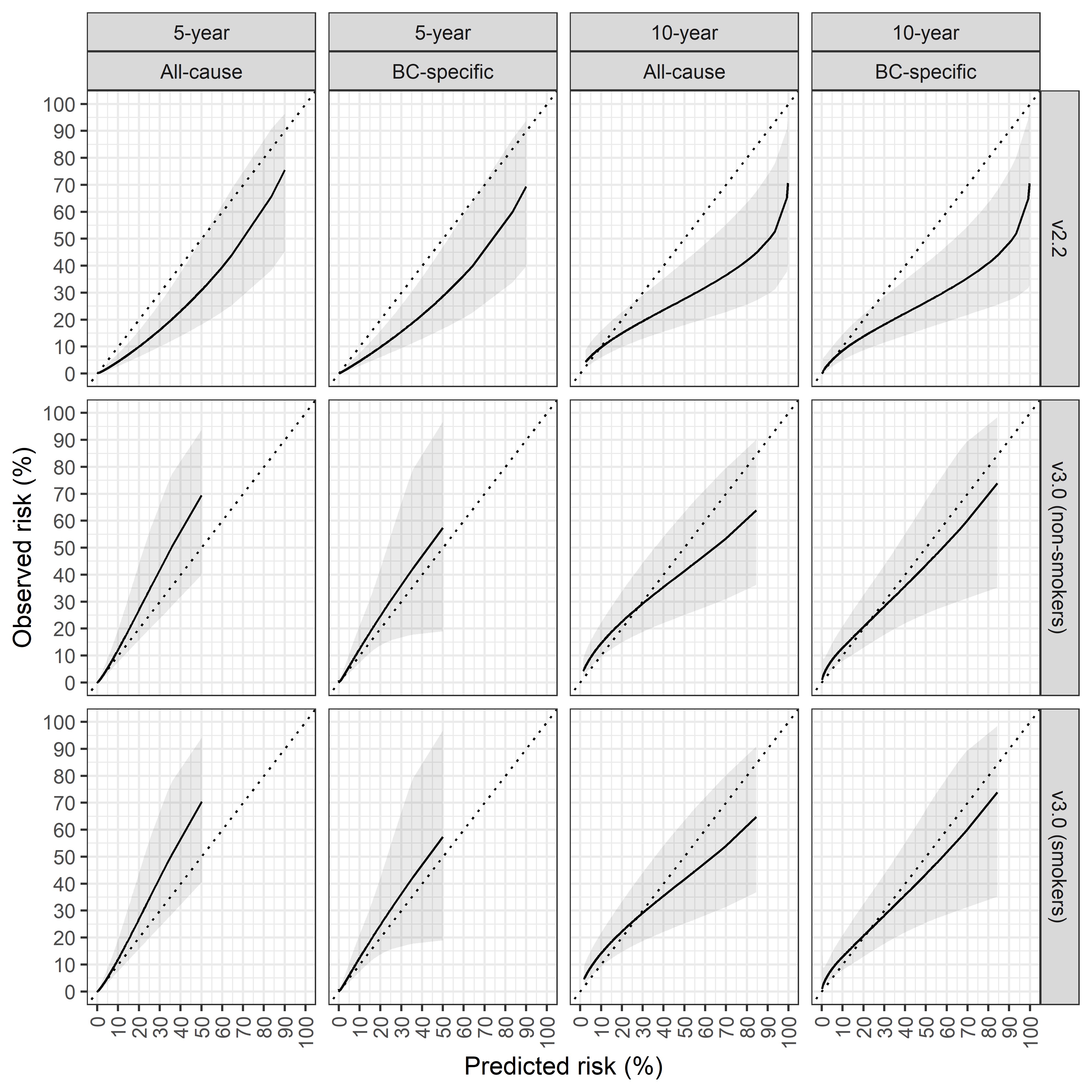


**Figure S2.** Calibration plots for PREDICT v2.2 and v3.0 in Luminal patients

Observed and predicted risks (%) for patients with Luminal tumors with respect to both all-cause and breast cancer-specific survival. V3.0 is illustrated assuming all patients to be smokers or non-smokers.

Abbreviations: BC, breast cancer


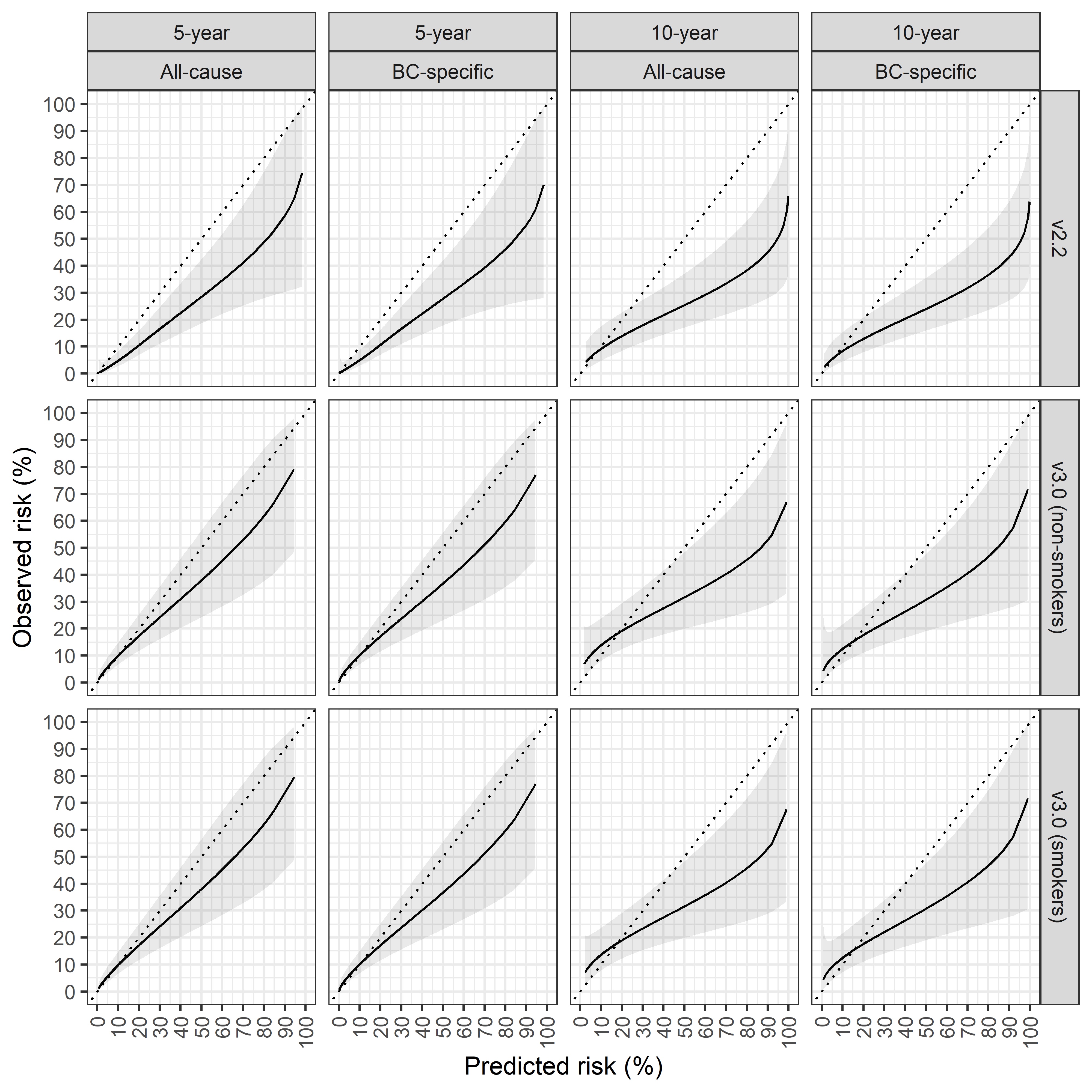


**Figure S3.** Calibration plots for PREDICT v2.2 and v3.0 in node-positive patients

Observed and predicted risks (%) for node-positive patients with respect to both all-cause and breast cancer-specific survival. V3.0 is illustrated assuming all patients to be smokers or non-smokers.

Abbreviations: BC, breast cancer


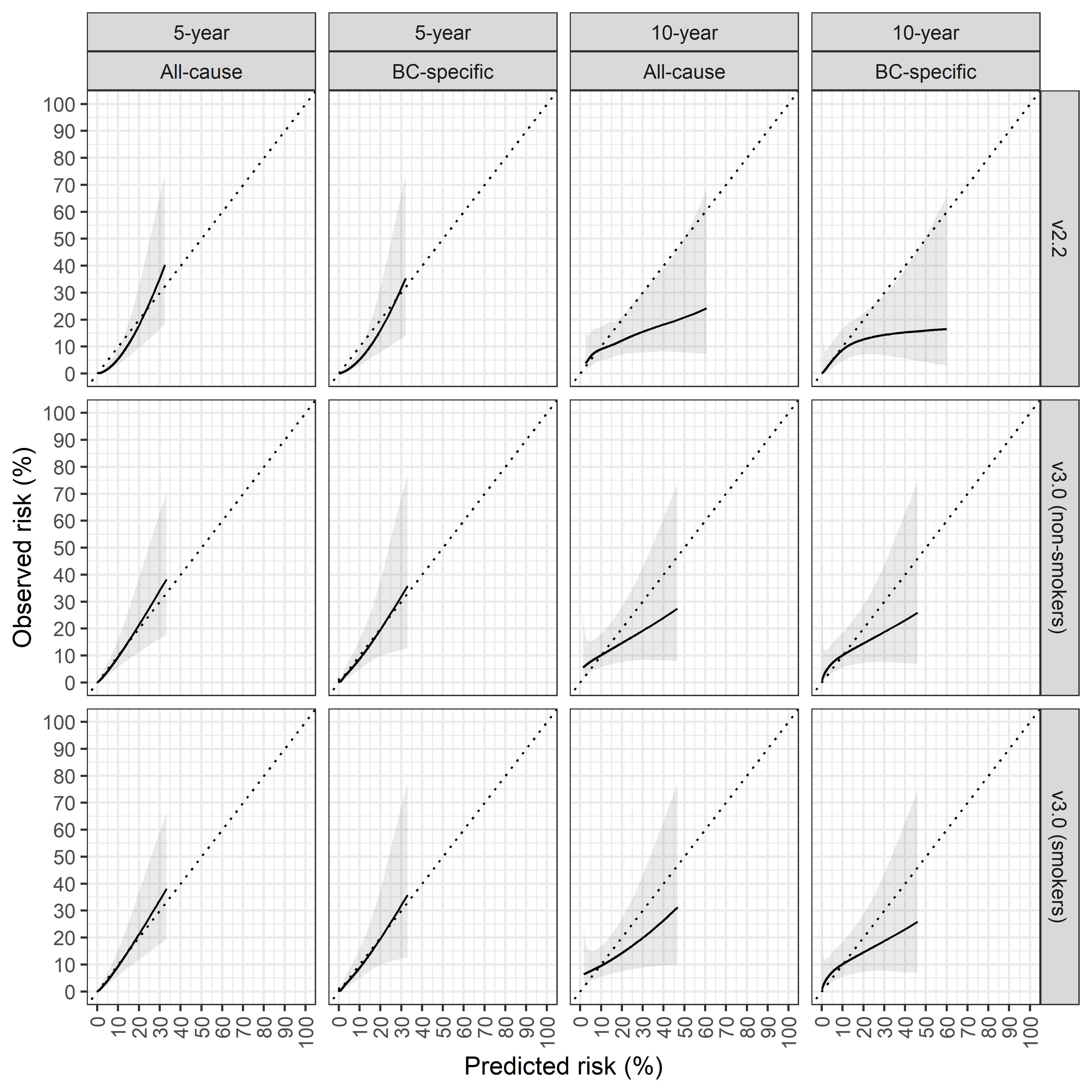


**Figure S4.** Calibration plots for PREDICT v2.2 and v3.0 in node-negative patients

Observed and predicted risks (%) for node-negative patients with respect to both all-cause and breast cancer-specific survival. V3.0 is illustrated assuming all patients to be smokers or non-smokers.

Abbreviations: BC, breast cancer


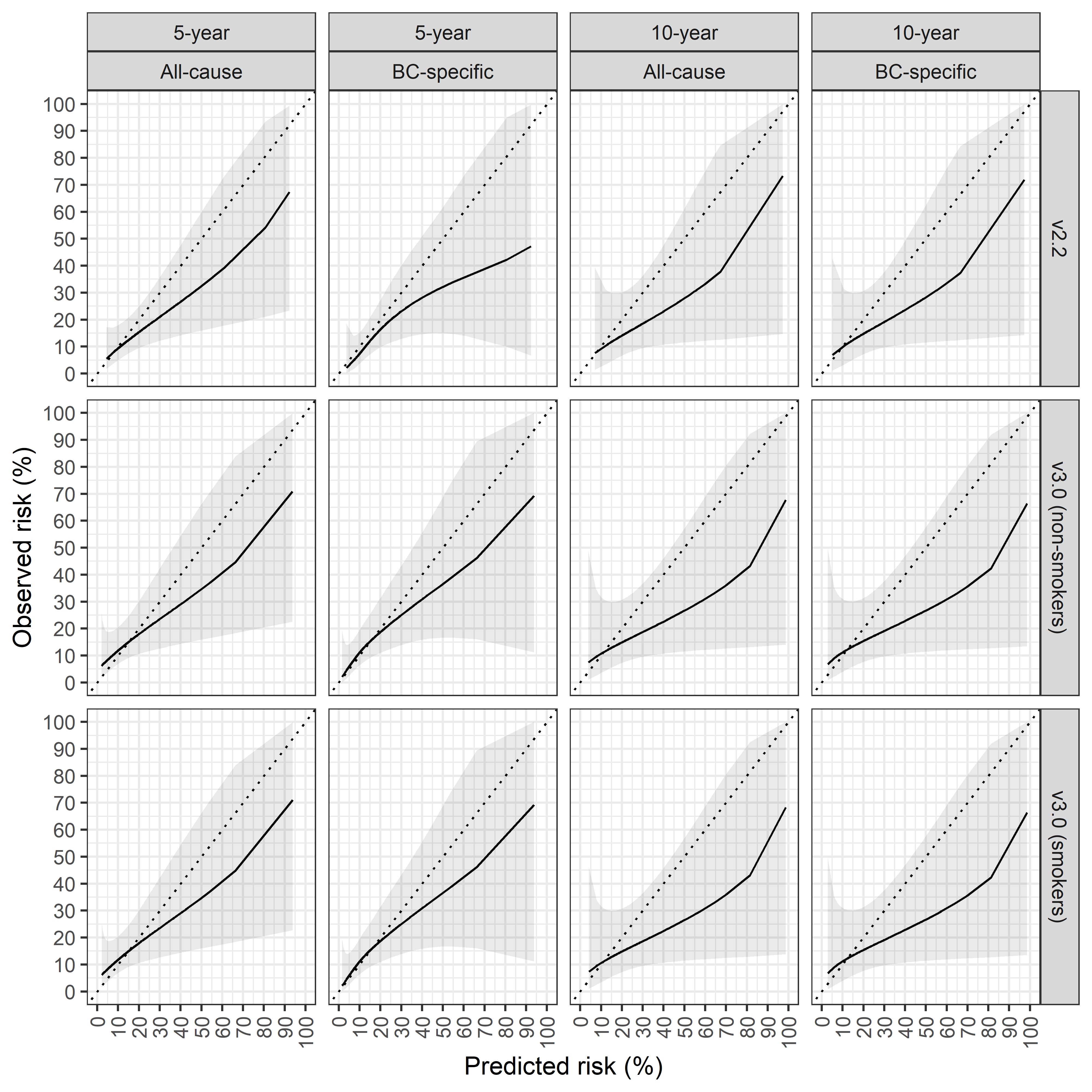


**Figure S5.** Calibration plots for PREDICT v2.2 and v3.0 in TNBC patients

Observed and predicted risks (%) for patients with TNBC tumors with respect to both all-cause and breast cancer-specific survival. V3.0 is illustrated assuming all patients to be smokers or non-smokers.

Abbreviations: BC, breast cancer; TNBC, triple-negative breast cancer
